# “Stealing fire or stacking knowledge” by machine intelligence to model link prediction in complex networks

**DOI:** 10.1016/j.isci.2022.105697

**Published:** 2022-11-30

**Authors:** Alessandro Muscoloni, Carlo Vittorio Cannistraci

**Affiliations:** 1 Center for Complex Network Intelligence (CCNI), Tsinghua Laboratory of Brain and Intelligence (THBI), Tsinghua University, 160 Chengfu Road, SanCaiTang Building, Haidian District, Beijing 100084, China; 2 Department of Computer Science, Tsinghua University, Beijing, China; 3 Department of Biomedical Engineering, Tsinghua University, Beijing, China

**Keywords:** Artificial intelligence, Network

## Abstract

Current methodologies to model connectivity in complex networks either rely on network scientists’ intelligence to discover reliable physical rules or use artificial intelligence (AI) that stacks hundreds of inaccurate human-made rules to make a new one that optimally summarizes them together. Here, we provide an accurate and reproducible scientific analysis showing that, contrary to the current belief, stacking more good link prediction rules does not necessarily improve the link prediction performance to nearly optimal as suggested by recent studies. Finally, under the light of our novel results, we discuss the pros and cons of each current state-of-the-art link prediction strategy, concluding that none of the current solutions are what the future might hold for us. Future solutions might require the design and development of next generation “creative” AI that are able to generate and understand complex physical rules for us.

## Highlights

• A simple network intelligence rule outperforms stacking link prediction models
• Stacking more good methods does not improve the performance to nearly optimal
• Ensemble meta-learning does not currently show any clear advantage on human design
• Link prediction evaluation is improved by introducing corrections and innovations

## Graphical abstract

**Figure:**
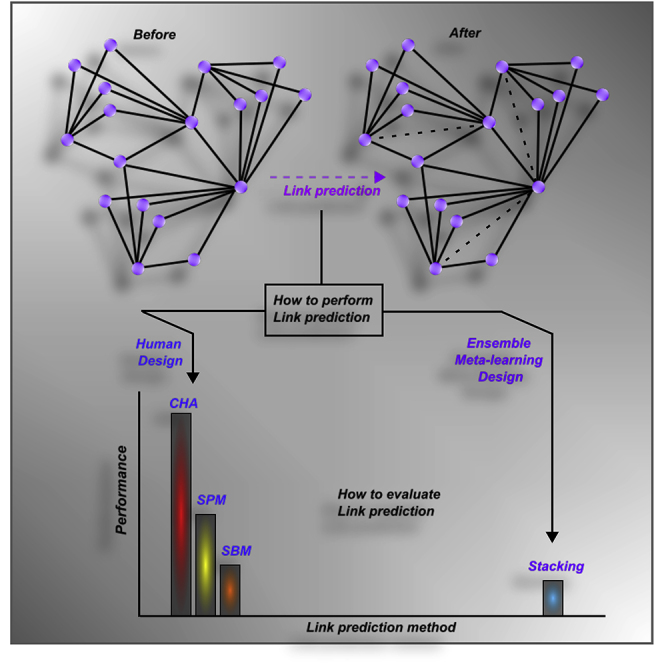

## Introduction

From nests to nets intricate wiring diagrams surround the birth and death of life and we, as humans, wonder what the secret rules behind such elegant network architectures are. If nature adopts connectivity to shape complexity, humans might adopt their intelligence, or an artificial one, to make sense of it. To predict structural complexity in a network, should we act as small Prometheans, exploiting creativity to “steal the fire from Gods” and search, as physicists aim, for that accurate but simple, elegant unique rule that makes sense of “everything”? Or should we ask artificial intelligence (AI) to stack for us hundreds of inaccurate human-made rules to make a new one that optimally summarizes them together? Perhaps, none of these two solutions is what the future holds for us.

Recently, Ghasemian et al.^1^ embraced the second option and proposed a stacking model: a machine learning method for link prediction in complex networks based on ensemble meta-learning, using artificial intelligence to create a single meta-model from hundreds of other models. The supervised algorithm learns from data the errors made by the individual predictors and decides how to combine them into a single predictor.^1^ Analyzing link prediction results on 550 real-world networks, the study concluded that the proposed stacking model for meta-learning is a state-of-the-art algorithm achieving nearly optimal prediction.^1^ However, the scientific community might benefit to interpret the results of stacking meta-learning^1^ when it is compared with other state-of-the-art algorithms that offer competitive performance according to computational strategies which largely differ from ensemble meta-learning.

An example of a completely different approach is given by the structural predictability method (SPM),^2^ an elegant model-free algorithm relying on a theory derived from quantum mechanics that exploits the first-order perturbation of the graph adjacency matrix to compute the inherent predictability of the network. SPM takes a way opposite to that of stacking: it simply does not assume any model. Studies have confirmed that SPM is among the best-performing state-of-the-art global approaches for topological link prediction.^2^^,^^3^

Another family of approaches for modeling and inference on networks is based on the statistical mechanics of complex systems.^4^^,^^5^^,^^6^ In particular, a widely developed framework for statistical analysis on networks is the stochastic block model (SBM), also adopted for prediction tasks such as community detection and link prediction.^7^ The model considers the nodes partitioned into blocks with certain probabilities of links existing between nodes of each block. The inference is generally performed using maximum likelihood optimization in order to find the most plausible partitions of nodes.

Interestingly, also brain and life science theories contribute to inspire revolutionary algorithms for link prediction.^8^ While most local rules for modeling connectivity were based on paths of length 2, Daminelli et al.^9^ and Kovács et al.^10^ elucidated the importance to build also models that rely on paths of length 3, inducing quadrangular closure.^9^ Thereafter, Muscoloni et al.^11^ proposed a general theory of network intelligence which is based on adaptive network automata, including a simple modeling rule named Cannistraci-Hebb (CH). CH is inspired by neuroplasticity concepts and scales adaptively on paths of different length (such as length 2 and 3), in relation with the intrinsic network structure.^11^

## Results

**Figure 1:**
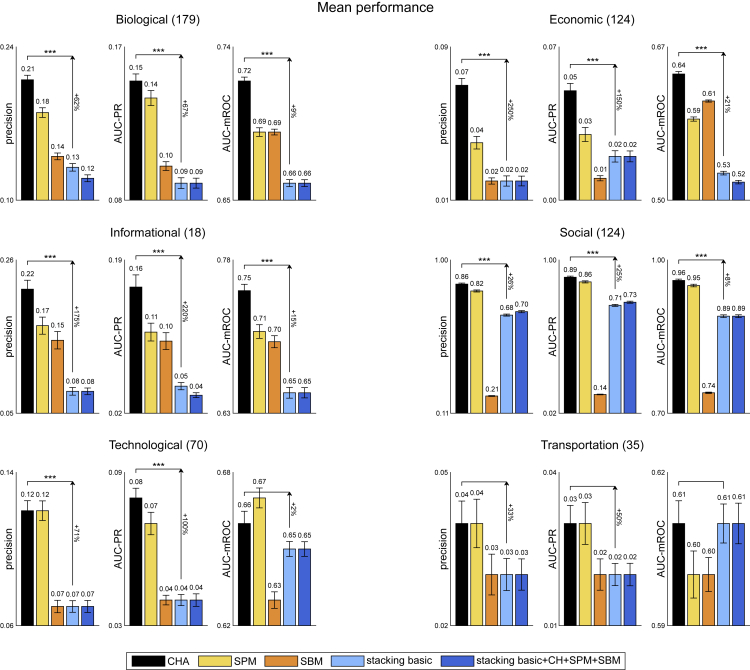
Mean performance evaluation of link prediction 550 real-world networks of Ghasemian et al.^1^ are considered. For each network and for each link prediction method, the link prediction evaluation framework is applied (10 repetitions). The barplots report, for each network domain (biological, economic, informational, social, technological, and transportation) and for each evaluation measure (precision, AUC-PR, and AUC-mROC), the mean performance of each method over the networks of that domain and over the 10 repetitions. The arrows highlight the percentage of improvement between CHA and stacking basic as $\frac{\left|CHA-stacking\right|}{\min\left(CHA,stacking\right)}\cdot 100$. The asterisks represent the p value significance of the permutation test for the mean, comparing the performances of CHA and stacking basic on the networks of that domain: one (∗), two (∗∗), or three (∗∗∗) asterisks depending on whether the p value is lower than or equal to the significance thresholds 0.05, 0.01, or 0.001, respectively. The number of networks of each domain is indicated in brackets. Error bars show the standard error of the mean, and the mean performance is reported on top of them.

**Figure 2:**
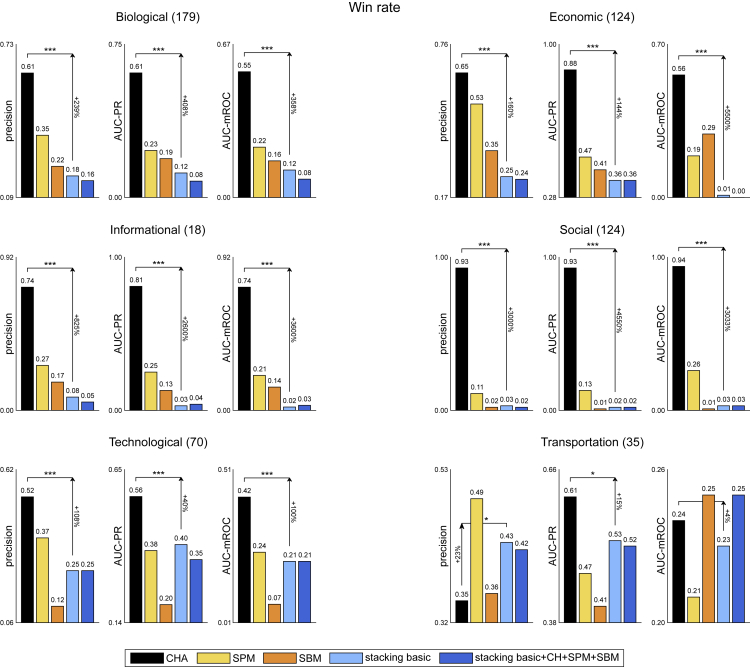
Win rate evaluation of link prediction The barplots report, for each network domain (biological, economic, informational, social, technological, and transportation) and for each evaluation measure (precision, AUC-PR, and AUC-mROC), the win rate of each method over the networks that domain and over the 10 repetitions. The win rate is the proportion of networks and repetitions in which the method obtains the best performance among all the methods, according to a certain evaluation measure. The value is shown on top of the bar. The arrows highlight the percentage of improvement between CHA and stacking basic as $\frac{\left|CHA-stacking\right|}{\min\left(CHA,stacking\right)}\cdot 100$. The asterisks represent the p value significance of the permutation test for the win rate, comparing the wins and losses of CHA and stacking basic on the networks of that domain: one (∗), two (∗∗), or three (∗∗∗) asterisks depending on whether the p value is lower than or equal to the significance thresholds 0.05, 0.01, or 0.001, respectively. The number of networks of each domain is indicated in brackets.

It might be enlightening to compare a method that stacks multiple models versus: (1) SPM, which is model-free and inspired by first-order perturbation theory in quantum mechanics; (2) SBM, which is based on a statistical mechanics modeling; (3) CH-adaptive (CHA), which relies on one model adapting to the intrinsic network structure. Indeed, in this study, using the 550 networks of Ghasemian et al.,^1^ we perform an accurate and reproducible scientific analysis reporting the results according to mean performance (Figure 1, Table 1) and win rate (Figure 2, Table 2) for several evaluation measures, following evaluation strategies previously employed^2^^,^^12^ (see Methods section for details).

**Table 1:** Mean performance evaluation of link prediction

		CHA	SPM	SBM	stacking basic	stacking basic + CH + SPM + SBM
**Precision**	Biological	**0.205 ± 0.004**	0.180 ± 0.004	0.141 ± 0.003	0.129 ± 0.003	0.115 ± 0.003
	Economic	**0.068 ± 0.004**	0.043 ± 0.003	0.021 ± 0.002	0.023 ± 0.003	0.020 ± 0.003
	Informational	**0.217 ± 0.012**	0.169 ± 0.012	0.152 ± 0.012	0.077 ± 0.005	0.075 ± 0.005
	Social	**0.857 ± 0.006**	0.822 ± 0.006	0.209 ± 0.003	0.684 ± 0.006	0.701 ± 0.006
	Technological	**0.124 ± 0.005**	0.121 ± 0.005	0.071 ± 0.003	0.073 ± 0.003	0.070 ± 0.003
	Transportation	0.036 ± 0.004	**0.042 ± 0.005**	0.032 ± 0.004	0.034 ± 0.003	0.033 ± 0.003
	*Mean*	**0.251 ± 0.125**	0.230 ± 0.121	0.104 ± 0.030	0.170 ± 0.104	0.169 ± 0.107
**AUC-precision**	Biological	**0.273 ± 0.006**	0.230 ± 0.006	0.176 ± 0.004	0.158 ± 0.005	0.138 ± 0.004
	Economic	**0.096 ± 0.005**	0.067 ± 0.004	0.025 ± 0.002	0.028 ± 0.003	0.025 ± 0.003
	Informational	**0.306 ± 0.017**	0.236 ± 0.014	0.191 ± 0.012	0.101 ± 0.009	0.096 ± 0.007
	Social	**0.922 ± 0.006**	0.909 ± 0.006	0.260 ± 0.003	0.770 ± 0.006	0.772 ± 0.007
	Technological	**0.179 ± 0.008**	0.175 ± 0.008	0.092 ± 0.004	0.103 ± 0.005	0.093 ± 0.004
	Transportation	**0.056 ± 0.007**	**0.056 ± 0.007**	0.040 ± 0.006	0.043 ± 0.005	0.043 ± 0.004
	*Mean*	**0.305 ± 0.130**	0.279 ± 0.130	0.131 ± 0.038	0.200 ± 0.115	0.195 ± 0.117
**AUC-PR**	Biological	**0.154 ± 0.004**	0.136 ± 0.004	0.099 ± 0.003	0.095 ± 0.003	0.087 ± 0.003
	Economic	**0.045 ± 0.003**	0.029 ± 0.003	0.012 ± 0.001	0.018 ± 0.003	0.017 ± 0.002
	Informational	**0.158 ± 0.013**	0.111 ± 0.010	0.099 ± 0.009	0.045 ± 0.004	0.043 ± 0.003
	Social	**0.885 ± 0.007**	0.862 ± 0.007	0.136 ± 0.002	0.712 ± 0.007	0.725 ± 0.007
	Technological	**0.077 ± 0.004**	0.073 ± 0.004	0.037 ± 0.002	0.038 ± 0.002	0.037 ± 0.002
	Transportation	**0.027 ± 0.004**	0.026 ± 0.004	0.019 ± 0.003	0.019 ± 0.002	0.019 ± 0.002
	*Mean*	**0.224 ± 0.134**	0.206 ± 0.132	0.067 ± 0.021	0.155 ± 0.112	0.155 ± 0.115
**AUCROC**	Biological	0.891 ± 0.002	0.778 ± 0.004	**0.905 ± 0.001**	0.864 ± 0.003	0.866 ± 0.003
	Economic	0.805 ± 0.002	0.530 ± 0.004	**0.905 ± 0.002**	0.699 ± 0.003	0.665 ± 0.004
	Informational	0.875 ± 0.007	0.806 ± 0.009	**0.886 ± 0.006**	0.858 ± 0.009	0.866 ± 0.009
	Social	**0.983 ± 0.002**	0.974 ± 0.002	0.964 ± 0.001	0.981 ± 0.002	0.981 ± 0.002
	Technological	0.816 ± 0.004	0.712 ± 0.005	0.847 ± 0.004	**0.854 ± 0.004**	0.847 ± 0.004
	Transportation	**0.847 ± 0.007**	0.659 ± 0.007	0.837 ± 0.004	0.837 ± 0.008	0.810 ± 0.009
	*Mean*	0.870 ± 0.026	0.743 ± 0.061	**0.891 ± 0.019**	0.849 ± 0.037	0.839 ± 0.042
**AUC-mROC**	Biological	**0.719 ± 0.002**	0.690 ± 0.003	0.685 ± 0.002	0.663 ± 0.002	0.655 ± 0.002
	Economic	**0.643 ± 0.003**	0.591 ± 0.002	0.606 ± 0.001	0.527 ± 0.002	0.522 ± 0.002
	Informational	**0.746 ± 0.006**	0.707 ± 0.007	0.700 ± 0.006	0.648 ± 0.005	0.649 ± 0.005
	Social	**0.963 ± 0.003**	0.953 ± 0.003	0.744 ± 0.001	0.890 ± 0.003	0.887 ± 0.003
	Technological	0.659 ± 0.005	**0.670 ± 0.004**	0.635 ± 0.003	0.650 ± 0.003	0.646 ± 0.003
	Transportation	**0.613 ± 0.004**	0.605 ± 0.005	0.597 ± 0.003	0.611 ± 0.004	0.608 ± 0.004
	*Mean*	**0.724 ± 0.052**	0.703 ± 0.054	0.661 ± 0.024	0.665 ± 0.049	0.661 ± 0.050
**NDCG**	Biological	**0.556 ± 0.004**	0.513 ± 0.005	0.509 ± 0.003	0.482 ± 0.004	0.473 ± 0.004
	Economic	**0.377 ± 0.004**	0.306 ± 0.004	0.326 ± 0.003	0.272 ± 0.004	0.269 ± 0.004
	Informational	**0.592 ± 0.011**	0.537 ± 0.012	0.531 ± 0.011	0.463 ± 0.009	0.464 ± 0.008
	Social	**0.952 ± 0.004**	0.946 ± 0.004	0.653 ± 0.002	0.907 ± 0.004	0.904 ± 0.004
	Technological	**0.477 ± 0.006**	0.465 ± 0.006	0.437 ± 0.005	0.445 ± 0.004	0.441 ± 0.004
	Transportation	**0.393 ± 0.008**	0.376 ± 0.008	0.368 ± 0.006	0.388 ± 0.007	0.384 ± 0.007
	*Mean*	**0.558 ± 0.086**	0.524 ± 0.092	0.471 ± 0.049	0.493 ± 0.088	0.489 ± 0.088
**MCC**	Biological	**0.200 ± 0.004**	0.175 ± 0.004	0.135 ± 0.003	0.122 ± 0.003	0.109 ± 0.003
	Economic	**0.067 ± 0.004**	0.042 ± 0.003	0.020 ± 0.002	0.022 ± 0.003	0.019 ± 0.002
	Informational	**0.215 ± 0.012**	0.167 ± 0.012	0.149 ± 0.012	0.074 ± 0.005	0.073 ± 0.005
	Social	**0.855 ± 0.006**	0.821 ± 0.006	0.206 ± 0.003	0.683 ± 0.006	0.699 ± 0.006
	Technological	**0.123 ± 0.005**	0.120 ± 0.005	0.070 ± 0.003	0.072 ± 0.003	0.069 ± 0.003
	Transportation	0.036 ± 0.004	**0.042 ± 0.005**	0.031 ± 0.004	0.033 ± 0.003	0.032 ± 0.003
	*Mean*	**0.249 ± 0.125**	0.228 ± 0.121	0.102 ± 0.030	0.168 ± 0.104	0.167 ± 0.107

550 real-world networks of Ghasemian et al.^1^ are considered. For each network and for each link prediction method, the link prediction evaluation framework is applied (10 repetitions). The table reports, for each network domain and for each evaluation measure, the mean performance, and standard error of the mean of each method over the networks of that domain and over the 10 repetitions. For each evaluation measure and for each link prediction method, the mean and standard error over the network domains is also reported. For each evaluation measure and for each domain, the highest mean result is highlighted in bold, as well as the highest overall mean result over the domains.

**Table 2:** Win rate evaluation of link prediction

		CHA	SPM	SBM	stacking basic	stacking basic + CH + SPM + SBM
**precision**	Biological	**0.607**	0.353	0.223	0.179	0.158
	Economic	**0.653**	0.527	0.354	0.249	0.244
	Informational	**0.744**	0.272	0.167	0.083	0.050
	Social	**0.932**	0.111	0.022	0.027	0.019
	Technological	**0.521**	0.366	0.121	0.246	0.253
	Transportation	0.349	**0.491**	0.360	0.434	0.423
	*Mean*	**0.634**	0.353	0.208	0.203	0.191
**AUC precision**	Biological	**0.542**	0.271	0.161	0.152	0.133
	Economic	**0.669**	0.556	0.332	0.278	0.275
	Informational	**0.656**	0.311	0.117	0.061	0.056
	Social	**0.938**	0.244	0.016	0.031	0.027
	Technological	**0.480**	0.343	0.136	0.233	0.234
	Transportation	0.414	**0.434**	0.377	0.394	0.411
	*Mean*	**0.616**	0.360	0.190	0.192	0.189
**AUC-PR**	Biological	**0.611**	0.234	0.193	0.118	0.082
	Economic	**0.877**	0.468	0.410	0.364	0.362
	Informational	**0.811**	0.250	0.133	0.033	0.039
	Social	**0.932**	0.134	0.015	0.023	0.019
	Technological	**0.561**	0.379	0.200	0.401	0.350
	Transportation	**0.606**	0.471	0.414	0.529	0.517
	*Mean*	**0.733**	0.323	0.228	0.245	0.228
**AUC-ROC**	Biological	**0.407**	0.158	0.322	0.182	0.322
	Economic	0.078	0.028	**0.900**	0.040	0.040
	Informational	0.367	0.044	**0.483**	0.189	0.339
	Social	**0.927**	0.411	0.026	0.904	0.905
	Technological	0.129	0.007	0.360	0.377	**0.440**
	Transportation	0.417	0.006	0.214	**0.460**	0.274
	*Mean*	**0.387**	0.109	0.384	0.359	**0.387**
**AUC-mROC**	Biological	**0.550**	0.223	0.165	0.116	0.079
	Economic	**0.557**	0.192	0.289	0.006	0.003
	Informational	**0.744**	0.206	0.139	0.022	0.028
	Social	**0.944**	0.259	0.015	0.026	0.025
	Technological	**0.421**	0.236	0.066	0.213	0.210
	Transportation	0.237	0.214	**0.251**	0.226	**0.251**
	*Mean*	**0.576**	0.222	0.154	0.101	0.099
**NDCG**	Biological	**0.551**	0.215	0.164	0.099	0.069
	Economic	**0.577**	0.117	0.368	0.005	0.002
	Informational	**0.800**	0.200	0.117	0.017	0.022
	Social	**0.932**	0.460	0.015	0.046	0.037
	Technological	**0.443**	0.170	0.057	0.260	0.231
	Transportation	**0.297**	0.154	0.243	0.294	0.271
	*Mean*	**0.600**	0.219	0.161	0.120	0.105
**MCC**	Biological	**0.607**	0.353	0.223	0.179	0.158
	Economic	**0.653**	0.526	0.354	0.250	0.244
	Informational	**0.744**	0.272	0.167	0.083	0.050
	Social	**0.931**	0.112	0.022	0.027	0.019
	Technological	**0.521**	0.369	0.124	0.249	0.257
	Transportation	0.349	**0.491**	0.360	0.434	0.423
	*Mean*	**0.634**	0.354	0.208	0.204	0.192

550 real-world networks of Ghasemian et al.^1^ are considered. For each network and for each link prediction method, the link prediction evaluation framework is applied (10 repetitions). The table reports, for each network domain and for each evaluation measure, the win rate of each method over the networks of that domain and over the 10 repetitions. For each evaluation measure and for each link prediction method, the mean over the network domains is also reported. For each evaluation measure and for each domain, the highest mean result is highlighted in bold, as well as the highest overall mean result over the domains.

The conclusion is that brute-force stacking of numerous algorithms by ensemble meta-learning AI does not perform better than (and is often significantly outperformed by) one simple brain-bioinspired rule such as CH. Also SPM and SBM seem generally to perform better than stacking. When we consider mean performance (Figure 1) for 3 evaluation measures (precision, AUC-PR, and AUC-mROC) and 6 network domains (18 total comparisons), CHA significantly outperforms stacking in 14/18 comparisons (with median improvement of 64.5%) and stacking never significantly outperforms CHA. If we consider win rate performance (Figure 2), CHA significantly outperforms stacking in 16/18 comparisons (with median improvement of 383%) and stacking significantly outperforms CHA in 1/18 comparisons (with improvement of 23%).

The result of this analysis agrees with the Gödel incompleteness: stacking might be nearly optimal but incomplete, by stacking you cannot squeeze out more than what is in your features and their interaction. To overcome this limitation, we ran comparisons including in the stacking procedure SPM, SBM, and each CH-model adopted in CHA (stacking basic + CH + SPM + SBM, in Figures 1 and 2). In particular, we stacked the scores of 8 CH-models (RA-L2, RA-L3, CH1-L2, CH1-L3, CH2-L2, CH2-L3, CH3-L2, and CH3-L3) and the 8 related CH-SPcorr scores,^11^ for a total of 16 CH features included in the stacking algorithm, in addition to 1 SPM feature and 1 SBM feature (see Methods section for details). Differently from the conclusion of Ghasemian et al. that stacking more good methods would improve the performance to nearly optimal, we found that, overall, there is no clear advantage in adding CH, SPM, and SBM to the basic stacking. Indeed, stacking basic and stacking basic + CH + SPM + SBM show comparable performances over different network domains and evaluation measures. However, if we look closer at the results of Ghasemian et al.,^1^ this is not totally unexpected. Let’s consider Table 1 of Ghasemian et al.,^1^ which reports the link prediction performance of individual algorithms and of stacking models on the 550 real-world networks, evaluated using AUC-ROC, precision, and recall. For precision, which is the most appropriate to evaluate link prediction among the three measures reported (as we will comment later), the best individual predictor is S-NB^13^ (spectral method with non-backtracking matrix), with a mean precision of 0.32. On the other side, stacking all the model-based predictors (including S-NB) leads to a mean precision of 0.25. In particular, all the stacking variants reported in Table 1 of Ghasemian et al.^1^ (including topological, model-based, and embedding-based predictors) have a mean precision lower than the one of the best individual predictor (S-NB). The main claims of Ghasemian et al.^1^ about near optimality are based on evaluations using AUC-ROC, although several recent studies have shown that AUC-ROC is inappropriate for early retrieval problems and for unbalanced prediction tasks,^9^^,^^14^^,^^15^^,^^16^^,^^17^^,^^18^^,^^19^^,^^20^ such as link prediction. A recent study of Muscoloni and Cannistraci^21^ proposes the AUC-mROC that we report in the main figures of this study (in lieu of AUC-ROC whose values are provided for completeness in Tables 1 and 2 of this study), which adjusts the evaluation issues largely commented in the literature about the AUC-ROC. In conclusion, the precision-based results in Table 1 of Ghasemian et al.,^1^ as well as the results in our study, are rising concrete concerns about the near optimality of stacking models when the link prediction task is evaluated according to more appropriate evaluation measures.

In Tables 1 and 2, we report the same results as Figures 1 and 2 including AUC-ROC and some additional evaluation measures (AUC-precision, NDCG, and MCC) as well as the mean over the network domains. The rationale to introduce these other evaluation measures is the following. The area under the precision curve (AUC-precision) was adopted in previous studies of network-based prediction of protein interaction in order to overcome the limitations of AUC-ROC deceptiveness in link prediction.^10^^,^^22^ The normalized discounted cumulative gain (NDCG) was proposed^23^^,^^24^ for assessment of algorithms in information retrieval of documents with the aim to address the inaptness of AUC-ROC in early retrieval problems such as link prediction. Therefore, as suggested by Tao Zhou in one of his recent reviews,^25^ we decided to apply NDCG also in link prediction evaluation. Finally, as sanity check that AUC-ROC evaluation is misleading, we consider a measure of binary classification known as the Matthews correlation coefficient (MCC).^26^^,^^27^ MCC is a binary classification rate that generates a high score only if the binary predictor was able to correctly predict most of positive data instances and most of negative data instances.^28^^,^^29^ This means that, differently from AUC-ROC, MCC provides a fair estimate of the predictor performance in class unbalanced datasets such as the one in link prediction problem. However, differently from the other early retrieval measures (precision, AUC-PR, AUC-precision, NDCG, and AUC-mROC) MCC does not give more importance to the positive class and it fairly and balanced considers the position in the ranking of positive and negative (in our case non-observed links) instances.

We can notice that all the evaluation measures except AUC-ROC provide coherent results, highlighting CHA as the method with the best mean value over the network domains (both for mean performance and win rate). AUC-ROC, instead, for some specific domains leads to a different conclusion, and indicates SBM as the best method for mean performance over the domains. The misleading evaluation performance of AUC-ROC that we notice in this large investigation agrees with recent studies reporting AUC-ROC as a deceptive measure for link prediction evaluation.^14^^,^^15^ However, our finding that MCC—which is designed as AUC-ROC to be a binary classification rate and not an early retrieval measure—disagrees with AUC-ROC and clearly agrees with the other early retrieval measures, provides an incontrovertible evidence that AUC-ROC is unreliable for evaluation of some link prediction scenario. This result is explained by the fact that MCC, as AUC-ROC, neglects the early retrieval nature of the problem but, differently from AUC-ROC, is able to adjust for the class unbalance. To understand better the scenarios in which AUC-ROC works and the ones in which it is misleading, we made another specific study that analyzes the misleading results of AUC-ROC and proposes the AUC-mROC as a new evaluation measure. AUC-mROC can adjust the AUC-ROC for evaluation of early retrieval problems in general and link prediction in particular.^21^ For this reason, we prefer to report AUC-mROC in the main figures of this study, and AUC-ROC in the Tables 1 and 2.

## Discussion

In conclusion, we might benefit to pursue new advanced strategies that overcome the current ones by means of “creative” AI that resembles human-like physical “understanding” of simple generalized rules associated with complexity.^30^ The methods and ways to design this next generation “creative” AI for complexity understanding are still under debate.^31^ The future might be populated by AI that “steals for us the fire from Gods”, toward machine intelligence that creates new rules, and then stack them with the ones already known.

### Limitations of the study

Although we have pointed out some problems with the stacking model, SPM, SBM, and CHA also have intrinsic limitations. SPM is a robust and purpose-wide algorithm because it is model-free and can be applied to any type of network regardless of the complex system rules that are behind the generation of the structural connectivity. However, if on one side the model-free strategy of SPM ensures good performance on many types of networks regardless of their origin, on the other side it cannot offer any hint on how to model the mechanisms and rules of the complex system which is behind the structural network dynamics. Both SBM and CH-modeling, together with its adaptive implementation called CHA, in part reply to this necessity to explicitly understand the mechanisms and rules of the complex system behind the network structure, but they are incomplete similarly to stacking model. This means that they might fail whenever the set of rules that they encode are not respected by a certain network topology. Furthermore, future studies might consider different types of prediction tasks for evaluation of link prediction. For instance, prediction of links on temporal networks, which means to predict the appearance of links in the future using a previous network structure; or prediction of links on synthetic networks,^32^^,^^33^ which allows us to investigate how the link predictors change their performance in relation to topological features that can be tuned by parameters in the generative synthetic network model.^32^^,^^33^

## STAR★Methods

### Key resources table

**Table:** 

REAGENT or RESOURCE	SOURCE	IDENTIFIER
**Software and algorithms**		

MATLAB 2020b	MathWorks	https://www.mathworks.com/
Python 3.9	Python Software Foundation	https://www.python.org/
CHA	Muscoloni et al.^11^	https://github.com/biomedical-cybernetics/stealing_fire_or_stacking_knowledge_to_model_link_prediction
SPM	Lü et al.^2^	https://github.com/biomedical-cybernetics/stealing_fire_or_stacking_knowledge_to_model_link_prediction
SBM	Peixoto^7^	https://graph-tool.skewed.de/; https://github.com/biomedical-cybernetics/stealing_fire_or_stacking_knowledge_to_model_link_prediction
Stacking basic	Ghasemian et al.^1^; This paper	https://github.com/Aghasemian/OptimalLinkPrediction; https://github.com/biomedical-cybernetics/stealing_fire_or_stacking_knowledge_to_model_link_prediction
Stacking basic + CH + SPM + SBM	This paper	https://github.com/biomedical-cybernetics/stealing_fire_or_stacking_knowledge_to_model_link_prediction
Evaluation measures	Muscoloni and Cannistraci^21^	https://github.com/biomedical-cybernetics/stealing_fire_or_stacking_knowledge_to_model_link_prediction; https://github.com/biomedical-cybernetics/AUC-mROC

**Other**		

Real-networks dataset	Ghasemian et al.^1^; This paper	https://github.com/Aghasemian/OptimalLinkPrediction; https://github.com/biomedical-cybernetics/stealing_fire_or_stacking_knowledge_to_model_link_prediction

### Resource availability

#### Lead contact

Further information and requests for resources should be directed to and will be fulfilled by the lead contact, Carlo Vittorio Cannistraci (kalokagathos.agon@gmail.com).

#### Materials availability

This study did not generate new unique reagents.

### Method details

#### Link prediction methods

##### Cannistraci-Hebb (CH) network automata

The Cannistraci-Hebb (CH) theory has been introduced as a revision of the local-community-paradigm (LCP) theory^8^^,^^9^^,^^20^^,^^34^ and it has been formalized within the framework of network automata.^11^ While the LCP paradigm emphasized the importance to complement the information related to the common neighbors with the interactions between them (internal local-community-links), the CH rule is based on the local isolation of the common neighbors by minimizing their interactions external to the local community (external local-community-links). In particular, Cannistraci-Hebb (CH) network automata on paths of length *n* are all the network automata models that explicitly consider the minimization of the external local-community-links within a local community characterized by paths of length *n*^11^.

We consider four CH network automata on paths of length 2 (L2), their mathematical formulas are^11^:

$$RA\_L2\left(u,v\right)=\sum_{z\in L2}\frac{1}{d_{z}}$$

$$CH1\_L2\left(u,v\right)=\sum_{z\in L2}\frac{di_{z}}{d_{z}}$$

$$CH2\_L2\left(u,v\right)=\sum_{z\in L2}\left(\frac{di_{z}^{*}}{de_{z}^{*}}=\frac{1+di_{z}}{1+de_{z}}\right)$$

$$CH3\_L2\left(u,v\right)=\sum_{z\in L2}\left(\frac{1}{de_{z}^{*}}=\frac{1}{1+de_{z}}\right)$$

where: *u* and *v* are the two seed nodes of the candidate interaction; z is the intermediate node on the considered path of length two; $d_{z}$ is the respective node degree; $di_{z}$ is the respective internal node degree; $de_{z}$ is the respective external node degree; and the summation is executed over all the paths of length two. The asterisk on a degree variable indicates that a unitary term is added, in order to avoid the saturation of the value.

We consider four CH network automata on paths of length 3 (L3), their mathematical formulas are^11^:

$$RA\_L3\left(u,v\right)=\sum_{z_{1},z_{2}\in L3}\frac{1}{\sqrt{d_{z_{1}}*d_{z_{2}}}}$$

$$CH1\_L3\left(u,v\right)=\sum_{z_{1},z_{2}\in L3}\frac{\sqrt{di_{z_{1}}*di_{z_{2}}}}{\sqrt{d_{z_{1}}*d_{z_{2}}}}$$

$$CH2\_L3\left(u,v\right)=\sum_{z_{1},z_{2}\in L3}\frac{\sqrt{di_{z_{1}}^{*}*di_{z_{2}}^{*}}}{\sqrt{de_{z_{1}}^{*}*de_{z_{2}}^{*}}}$$

$$CH3\_L3\left(u,v\right)=\sum_{z_{1},z_{2}\in L3}\frac{1}{\sqrt{de_{z_{1}}^{*}*de_{z_{2}}^{*}}}$$

where: *u* and *v* are the two seed nodes of the candidate interaction; $z_{1},z_{2}$ are the intermediate nodes on the considered path of length three; $d_{z_{1}},d_{z_{2}}$ are the respective node degrees; $di_{z_{1}},di_{z_{2}}$ are the respective internal node degreed; $de_{z_{1}},de_{z_{2}}$ are the respective external node degrees; and the summation is executed over all the paths of length three. The asterisk on a degree variable indicates that a unitary term is added, in order to avoid the saturation of the value.

##### Cannistraci-Hebb (CH) SPcorr

Given a network and the CH scores $CH_{i,j}$ already computed for all node pairs (i,j) according to a certain CH model, the shortest paths correlation score (SPcorr) is computed as follows^11^: (1) assign to each observed link (i,j) of the network a weight $w_{i,j}=\frac{1}{1+CH_{i,j}}$; (2) compute the weighted shortest path $SP_{i,j}$ for all node pairs (i,j) on the weighted network; (3) for each node pair (i,j), compute the prediction score $SPcorr_{i,j}$ as the Spearman’s rank correlation between the vector of all shortest paths from i, $SP_{i}=\left[SP_{i,1},SP_{i,2},\ldots ,SP_{i,N}\right]$, and the vector of all shortest paths from j, $SP_{j}=\left[SP_{j,1},SP_{j,2},\ldots ,SP_{j,N}\right]$.

##### Cannistraci-Hebb adaptive (CHA) network automaton

It has been shown that different complex networks can be organized according to different patterns of connectivity, such as L2 or L3, and therefore there is not a unique network automaton model that would be able to effectively describe all of them. For example, while performing link prediction on a social network one might decide to adopt a L2-based method, whereas on a PPI network it would likely be better to choose a L3-based method. The Cannistraci-Hebb Adaptive (CHA) network automaton has been designed to adapt to the network under investigation and to automatically select the model that would likely provide the best prediction.^11^

In order to do this, it exploits a particular property of the CH network automata models. Such deterministic rules for link prediction can assign both to observed and non-observed links a score that is comparable, meaning that the scores of observed links are not biased to be higher or lower than the scores of non-observed links. This is because the mathematical equation to compute the score of the connection between two nodes is independent from the existence of that link, whether the link is observed or not in the current topology does not affect the score.

Given a model, it is possible to assign likelihood scores to both observed and non-observed links and compute the AUC-PR to evaluate how well the model can discriminate them. The assumption is that, if the model tends to score observed links higher than non-observed links, then it would be more effective in predicting missing or future links. Therefore, the adaptive network automaton works as follows: given a network and a set of candidate models, for each model it computes the AUC-PR, then it automatically selects as link prediction result the scores of the non-observed links from the model that obtained the highest AUC-PR.

In this study, we considered the following CH models within the CHA network automaton: CH2-L2, CH3-L2, CH2-L3, CH3-L3. In the original study these models have been highlighted as the best set of models to adopt for building the adaptive version.^11^ In addition, each of the four CH models applied the associated CH-SPcorr score for sub-ranking, in order to internally sub-rank all the node pairs characterized by the same CH score, reducing the ranking uncertainty of node pairs that are tied-ranked.^11^ The MATLAB code of the CHA method and of the CH models used for the link prediction simulations have been developed by the authors and is publicly available at the GitHub repository associated to this study: https://github.com/biomedical-cybernetics/stealing_fire_or_stacking_knowledge_to_model_link_prediction.

##### Structural perturbation method (SPM)

The structural perturbation method (SPM) relies on a theory similar to the first-order perturbation in quantum mechanics.^2^ A high-level description of the procedure is the following: (1) randomly remove 10% of the links from the network adjacency matrix X, obtaining a reduced network X’ = X - R, where R is the set of removed links; (2) compute the eigenvalues and eigenvectors of X’; (3) considering the set of links R as a perturbation of X′, construct the perturbed matrix X^P^ via a first-order approximation that allows the eigenvalues to change while keeping fixed the eigenvectors; (4) repeat steps 1–3 for 10 independent iterations and take the average of the perturbed matrices X^P^. The link prediction result is given by the values of the average perturbed matrix, which represent the scores for each node pair. The higher the score the greater the likelihood that the interaction exists. The idea behind the method is that a missing part of the network is predictable if it does not significantly change the structural features of the observable part, represented by the eigenvectors of the matrix. If this is the case, the perturbed matrices should be good approximations of the original network.^2^ The MATLAB implementation of the SPM method has been provided by the authors of the original study^2^ and is publicly available at the GitHub repository associated to this study: https://github.com/biomedical-cybernetics/stealing_fire_or_stacking_knowledge_to_model_link_prediction.

##### Stochastic block model (SBM)

The general idea of stochastic block model (SBM) is that the nodes are partitioned into B blocks and a B x B matrix specifies the probabilities of links existing between nodes of each block. SBM provides a general framework for statistical analysis and inference in networks, in particular for community detection and link prediction.^7^ The concept of degree-corrected (DC) SBM has been introduced for community detection tasks^35^ and for prediction of spurious and missing links,^36^ in order to keep into account the variations in node degree typically observed in real networks. The implementation considered adopts an optimized Monte Carlo Markov Chain (MCMC) to sample the space of the possible partitions.^7^ In general the predictive performance is higher when averaging over collections of partitions than when considering only the single most plausible partition, since this can lead to overfitting.^37^ Therefore for a given network we sampled P partitions, for each partition we obtained the likelihood scores related to the non-observed links, and then considered the average likelihood scores as the link prediction result. We set p = 100 for networks with N ≤ 100, p = 50 for 100 < N ≤ 1000, p = 10 for N > 1000. The code of the SBM method has been released by the authors of the original study^7^ within the Graph-tool Python module available at: https://graph-tool.skewed.de/. The implementation used for the link prediction application is publicly available at the GitHub repository associated to this study: https://github.com/biomedical-cybernetics/stealing_fire_or_stacking_knowledge_to_model_link_prediction.

##### Stacking algorithm

Stacking is a meta-learning approach that can learn from data how to combine individual predictors into a single, potentially more accurate algorithm. It combines the predictors by learning a supervised model of input characteristics and the corresponding errors made by individual predictors.^1^

The version of the algorithm named “stacking basic” in the figures, which corresponds to the implementation released by the authors, contains 42 topological predictors as topological features. In particular, there are 8 network global features, 14 node-pair based features, 20 single-node based features. The complete list is provided in Table S1 of Ghasemian et al.^1^ The version of the algorithm named “stacking basic + CH + SPM + SBM” contains 60 topological features in total: the same 42 topological predictors of “stacking basic”, 16 CH-based features, 1 SPM score and 1 SBM score. In particular, the 16 CH-based features consists of the link prediction scores of the 8 CH models (RA-L2, CH1-L2, CH2-L2, CH3-L2, RA-L3, CH1-L3, CH2-L3, CH3-L3) in addition to the 8 SPcorr scores associated to the 8 CH models.

In the implementation released by the authors, random forest has been used as classifier. The best hyperparameters combination is chosen during cross-validation with a grid search on the following values: number of trees = [25, 50, 100], depth of trees = [3, 6]. The original Python code of the stacking model has been released by Ghasemian et al.^1^ at: https://github.com/Aghasemian/OptimalLinkPrediction. A customized version of the code for the algorithms named ““stacking basic” and “stacking basic + CH + SPM + SBM” is publicly available at the GitHub repository associated to this study: https://github.com/biomedical-cybernetics/stealing_fire_or_stacking_knowledge_to_model_link_prediction.

#### Link prediction evaluation framework

The 10% link removal evaluation framework is adopted when there is no information available about missing links or links that will appear in the future with respect to the time point of the network under consideration.

Given a network X, 10% of links are randomly removed, obtaining a reduced network X’ = X - R, where R is the set of removed links. For evaluating each algorithm, the reduced network X′ is given in input, obtaining in output a ranking of the non-observed links in X’ by decreasing likelihood scores. The prediction performance is evaluated considering as positive samples the set R of links previously removed, according to several evaluation measures: precision, area under precision curve (AUC-precision), area under precision-recall curve (AUC-PR), area under ROC curve (AUC-ROC), area under mROC curve (AUC-mROC), normalized discounted cumulative gain (NDCG), Matthews correlation coefficient (MCC). The reference articles to these evaluation measures are provided in the main text of the study. Due to the randomness of the link removal, the evaluation is repeated 10 times. The MATLAB code of the evaluation measures have been developed by the authors and is publicly available at the GitHub repository associated to this study: https://github.com/biomedical-cybernetics/stealing_fire_or_stacking_knowledge_to_model_link_prediction.

#### Real-networks dataset

The dataset consists of the 550 real-world networks adopted by Ghasemian et al.,^1^ which is publicly available at: https://github.com/Aghasemian/OptimalLinkPrediction and https://github.com/biomedical-cybernetics/stealing_fire_or_stacking_knowledge_to_model_link_prediction.The networks are divided into 6 different domains as follows: Biological (179), Economic (124), Informational (18), Social (124), Technological (70), Transportation (35). All networks are analyzed as undirected, unweighted, without self-loops and only using the largest connected component.

### Quantification and statistical analysis

#### Mean performance, win rate and permutation tests

The mean performance evaluation of link prediction is reported in Figure 1 and Table 1. For each network and for each link prediction method, the link prediction evaluation framework is applied (10 repetitions). Then, for each network domain and for each evaluation measure, we compute the mean performance and standard error of the mean of each method over the networks of that domain and over the 10 repetitions. For example, there are 179 networks in the Biological domain, therefore the mean performance and standard error of the mean are computed over 179 × 10 = 1790 values.

The win rate evaluation of link prediction is reported in Figure 2 and Table 2. The procedure is analogous to the one described above, except that the win rate is computed instead of the mean performance. The win rate is defined as the proportion of networks and repetitions (for example 179 × 10 = 1790 in the Biological domain) in which a certain method obtains the best performance among all the methods, according to a certain evaluation measure.

In order to assess if the mean performance of two methods is significantly different, we apply a permutation test of the mean. The results are shown for CHA versus stacking basic in Figure 1. For example, in the Biological domain and considering the precision as evaluation measure, the permutation test is applied on the 1790 precision values of CHA versus the 1790 precision values of stacking basic. The absolute difference of the two means of the two vectors is the observed statistic. For 1000 iterations, the two vectors are randomly permuted, and the same statistic is computed, obtaining a null distribution of statistics. Finally, the empirical p value is computed as the proportion of null statistics that are greater than or equal to the observed statistic.

In order to assess if the win rate of two methods is significantly different, we apply the same permutation test of the mean described above, but for each method the vector containing the performance values (for example of length 1790 in the Biological domain) is transformed into a binary vector representing the wins (value 1) or losses (value 0) of that method among all the methods, according to a certain evaluation measure. In practice, the mean of such binary vector is equal to the win rate of the method.

In Figures 1 and 2, as a result of these statistical tests comparing the performances of CHA versus stacking basic, we show one (∗), two (∗∗) or three (∗∗∗) asterisks depending on whether the p value is lower than or equal to the significance thresholds 0.05, 0.01 or 0.001 respectively.

## Data Availability

• This paper analyzes existing, publicly available data. The URLs are listed in the key resources table. • All original code has been deposited at GitHub and is publicly available as of the date of publication. The URLs are listed in the key resources table. • Any additional information required to reanalyze the data reported in this paper is available from the lead contact upon request.

## References

[1] Ghasemian A., Hosseinmardi H., Galstyan A., Airoldi E.M., Clauset A. (2020). Stacking models for nearly optimal link prediction in complex networks. Proc. Natl. Acad. Sci. USA.

[2] Lü L., Pan L., Zhou T., Zhang Y.-C., Stanley H.E. (2015). Toward link predictability of complex networks. Proc. Natl. Acad. Sci. USA.

[3] Wang W., Cai F., Jiao P., Pan L. (2016). A perturbation-based framework for link prediction via non-negative matrix factorization. Sci. Rep..

[4] Park J., Newman M.E.J. (2004). Statistical mechanics of networks. Phys. Rev. E Stat. Nonlin. Soft Matter Phys..

[5] Cimini G., Squartini T., Saracco F., Garlaschelli D., Gabrielli A., Caldarelli G. (2019). The statistical physics of real-world networks. Nat. Rev. Phys..

[6] De Domenico M., Biamonte J. (2016). Spectral entropies as information-theoretic tools for complex network comparison. Phys. Rev. X.

[7] Peixoto T.P. (2014). Efficient Monte Carlo and greedy heuristic for the inference of stochastic block models. Phys. Rev. E Stat. Nonlin. Soft Matter Phys..

[8] Cannistraci C.V. (2018). Modelling self-organization in complex networks via a brain-inspired network automata theory improves link reliability in protein interactomes. Sci. Rep..

[9] Daminelli S., Thomas J.M., Durán C., Vittorio Cannistraci C. (2015). Common neighbours and the local-community-paradigm for topological link prediction in bipartite networks. New J. Phys..

[10] Kovács I.A., Luck K., Spirohn K., Wang Y., Pollis C., Schlabach S., Bian W., Kim D.K., Kishore N., Hao T. (2019). Network-based prediction of protein interactions. Nat. Commun..

[11] Muscoloni, A., Michieli, U. & Cannistraci, C. V. Adaptive network automata modelling of complex networks. Preprints (2020). 10.20944/preprints202012.0808.v2

[12] Zhou T., Lee Y.-L., Wang G. (2021). Experimental analyses on 2-hop-based and 3-hop-based link prediction algorithms. Phys. Stat. Mech. Appl..

[13] Krzakala F., Moore C., Mossel E., Neeman J., Sly A., Zdeborová L., Zhang P. (2013). Spectral redemption in clustering sparse networks. Proc. Natl. Acad. Sci. USA.

[14] Lichtnwalter R., Chawla N.V. (2012). 2012 IEEE/ACM International Conference on Advances in Social Networks Analysis and Mining.

[15] Yang Y., Bao F., He Z., Hu J. (2015). Evaluating link prediction methods. Eur. J. Cardio. Thorac. Surg..

[16] Saito T., Rehmsmeier M. (2015). The precision-recall plot is more informative than the ROC plot when evaluating binary classifiers on imbalanced datasets. PLoS One.

[17] Clark R.D., Webster-Clark D.J. (2008). Managing bias in ROC curves. J. Comput. Aided Mol. Des..

[18] Swamidass S.J., Azencott C.-A., Daily K., Baldi P. (2010). A CROC stronger than ROC: measuring, visualizing and optimizing early retrieval. Bioinformatics.

[19] Truchon J.-F., Bayly C.I. (2007). Evaluating virtual screening methods: good and bad metrics for the ‘early recognition’ problem. J. Chem. Inf. Model..

[20] Durán C., Daminelli S., Thomas J.M., Haupt V.J., Schroeder M., Cannistraci C.V. (2017). Pioneering topological methods for network-based drug–target prediction by exploiting a brain-network self-organization theory. Brief. Bioinform..

[21] Muscoloni A., Cannistraci C.V. (2022). Early Retrieval Problem and Link Prediction Evaluation via the Area under the Magnified ROC. Preprints.

[22] Muscoloni A., Abdelhamid I., Cannistraci C.V. (2018). Local-community network automata modelling based on length-three-paths for prediction of complex network structures in protein interactomes, food webs and more. bioRxiv.

[23] Järvelin K., Kekäläinen J. (2000). *Proceedings of the 23rd Annual International ACM SIGIR Conference on Research and Development in Information Retrieval* 41–48.

[24] Järvelin K., Kekäläinen J. (2002). Cumulated gain-based evaluation of IR techniques. ACM Trans. Inf. Syst..

[25] Zhou T. (2021). Progresses and challenges in link prediction. iScience.

[26] Matthews B.W. (1975). Comparison of the predicted and observed secondary structure of T4 phage lysozyme. Biochim. Biophys. Acta.

[27] Yule G.U. (1912). On the methods of measuring association between two attributes. J. R. Stat. Soc..

[28] Jurman G., Riccadonna S., Furlanello C. (2012). A comparison of MCC and CEN error measures in multi-class prediction. PLoS One.

[29] Chicco D. (2017). Ten quick tips for machine learning in computational biology. BioData Min..

[30] Seif A., Hafezi M., Jarzynski C. (2021). Machine learning the thermodynamic arrow of time. Nat. Phys..

[31] Lemos P., Jeffrey N., Miles C., Ho S., Battaglia P. (2022). Rediscovering orbital mechanics with machine learning. arXiv.

[32] Muscoloni A., Cannistraci C.V. (2018). A nonuniform popularity-similarity optimization (nPSO) model to efficiently generate realistic complex networks with communities. New J. Phys..

[33] Alessandro M., Carlo Vittorio C. (2018). Leveraging the nonuniform PSO network model as a benchmark for performance evaluation in community detection and link prediction. New J. Phys..

[34] Cannistraci C.V., Alanis-Lobato G., Ravasi T. (2013). From link-prediction in brain connectomes and protein interactomes to the local-community-paradigm in complex networks. Sci. Rep..

[35] Karrer B., Newman M.E.J. (2011). Stochastic blockmodels and community structure in networks. Phys. Rev. E Stat. Nonlin. Soft Matter Phys..

[36] Zhang X., Wang X., Zhao C., Yi D., Xie Z. (2014). Degree-corrected stochastic block models and reliability in networks. Phys. Stat. Mech. Appl..

[37] Vallès-Català T., Peixoto T.P., Sales-Pardo M., Guimerà R. (2018). Consistencies and inconsistencies between model selection and link prediction in networks. Phys. Rev. E.

